# Zika virus testing of asymptomatic patients undergoing assisted reproduction in Curitiba, Brazil

**DOI:** 10.5935/1518-0557.20200063

**Published:** 2021

**Authors:** Ricardo Ditzel Delle Donne, Janaina de Almeida Furlan, Danilo Martins Rahal, Marilia Porto Bonow, Vinicius Bonato da Rosa, Alessandro Schuffner

**Affiliations:** 1 Master in Gynecology and Obstetrics from Federal University of Paraná (UFPR), Curitiba, PR, Brazil; 2 Assisted Reproduction Center of Guarapuava, Guarapuava, PR, Brazil; 3 Post Graduate Program in Gynecology and Obstetrics of Federal University of Paraná (UFPR), Curitiba, PR, Brazil; 4 Positivo University (UP), Curitiba, PR, Brazil; 5 Conceber Reproductive Medicine Center, Curitiba, PR, Brazil; 6 Master in Clinical Medicine from Federal University of Paraná (UFPR), Curitiba, PR, Brazil

**Keywords:** Zika virus, assisted reproduction, *in vitro* fertilization, intrauterine insemination

## Abstract

**Objective:**

The objective is to report recent data on the infection and detection of Zika virus in infertile couples and to discuss the need to make disease surveillance compulsory in this population in order to decrease the burden on the healthcare system and expedite treatment onset.

**Methods:**

We collected and analyzed the results of Zika virus infection screening tests of infertile couples in a private clinic in the low-incidence region of Curitiba - Brazil.

**Results:**

Among the 1189 serologies performed, 98.5% were negative for Zika virus, 0.75% were positive, and 0.75% were inconclusive. The twenty-one reverse transcription polymerase chain reaction tests performed for confirmation of infection were negative.

**Conclusion:**

Zika virus infection screening for asymptomatic patients may lead to delayed fertility treatment initiation in addition to excessive expenses for the patients. Based on our results, we challenge the validity of mandatory screening, especially in low-incidence regions.

## INTRODUCTION

Zika virus is a flavivirus initially identified only in mosquitoes in several African countries. In 1953, it was isolated in three Nigerian patients, representing the first record of the pathogen in humans. From this episode until 2007, when the first outbreak occurred in Micronesia, only 13 cases had been reported. Thereafter, other outbreaks occurred in the Pacific Islands. In March 2015, Zika virus was first identified in the Americas when an outbreak of exanthematous disease occurred in the state of Bahia and from there it spread throughout the Brazilian territory (Petersen *et al*., 2016).

Zika virus transmission occurs in most cases through bites of infected *Aedesaegypti* and *Aedesalbopictus* mosquitoes (Petersen *et al*., 2016). However, it appears that maternal-fetal transmission is also possible, because the virus has been isolated in the amniotic fluid and placentas from mothers of infants with microcephaly and in the brain tissue of fetuses carried by infected mothers (Calvet *et al*., 2016; Martines *et al*., 2016). In addition, transmission to women in non-endemic areas is thought to have occurred by sexual transmission after their male partners returned from trips to endemic areas (Hills *et al*., 2016).

Epidemiological bulletins released by the Brazilian Ministry of Health in 2016, during a Zika outbreak, and in 2017, after the outbreak had been controlled, reported that the incidence of Zika virus in Southern Brazil is low. In 2016, the South region had 109 confirmed cases, which was not very significant compared to the 30,683 nationwide confirmed cases that year. In 2017, the South region had 39 of the 316 nationwide confirmed cases (Brazilian Ministry of Health, 2016). In addition, an observational study indicated that in 2015, 1,608 cases of microcephaly were reported nationwide, of which only 27 cases (1.7%) occurred in the South (Marinho *et al*., 2016).

The incubation period for Zika virus is unknown, but is estimated to last approximately 7 days based on information from other flaviviruses (Pertersen *et al*., 2016). The most common symptoms identified during the outbreak in Micronesia were pruritic macular or papular skin rash, fever, arthritis or arthralgia, non-purulent conjunctivitis, myalgia, and headache (Duffy *et al*., 2009). However, the main symptom associated with the outbreak in Brazil in 2016 was microcephaly in infants and fetuses. The affected newborns presented with varying degrees of neurological and neuropsychomotor developmental alterations (Petersen *et al*., 2016).

Diagnosis of Zika virus infection can be made through reverse transcription polymerase chain reaction (RT-PCR) tests that detect the viral RNA (ribonucleic acid) or by detection of specific IgM and IgG antibodies in the serum. Detection of the virus by RT-PCR is possible only during the symptomatic period, when the virus is present in the bloodstream, which reduces the window of opportunity for detection. Immunological tests can detect signs of infection for longer periods, because IgM becomes positive approximately 1 week after infection and persists for several months (although the number of months has not yet been established) (Petersen *et al*., 2016).

The Food and Drug Administration (FDA) published a resolution with guidelines for Zika virus screening to reduce virus transmission in tissue and cell donors. Patients diagnosed with Zika virus infection, those having traveled to affected areas within the last 6 months, or women having had sexual intercourse with a man who had traveled to an affected area within the last 6 months should be considered ineligible for donation (FDA, 2016).

Given the alarming number of patients infected with Zika virus and the evidence for vertical transmission, the National Agency for Health Surveillance (ANVISA) published recommendations for germ cell and tissue banks. The new guidelines recommend that patients scheduled to undergo treatments involving manipulation of gametes (whether their own or from donors) should be serologically tested for Zika IgM antibodies. The test samples need to be collected no more than 5 days prior to the oocyte collection procedures. Only patients with negative test results can be referred for collection. In cases of inconclusive or positive results, the tests need to be repeated after 30 days for confirmation; alternatively, molecular tests for infection markers can be performed at any time (ANVISA, 2016).

Considering the low incidence in the South region, the objective of this communication was to report recent data on the infection and detection of Zika virus in infertile couples and to discuss the need to make disease surveillance compulsory in this population in order to decrease the burden on the healthcare system and expedite treatment onset.

## MATERIALS AND METHODS

The local ethics committee approved the study. Due to the retrospective nature of the study, patients were not required to give consent.

This was a descriptive, retrospective study involving the statistical analysis of screening tests results (serology and RT-PCR) for Zika virus in couples undergoing a cycle of assisted reproduction (*in vitro* fertilization or intrauterine insemination) at a private clinic in the city of Curitiba (State of Paraná, Brazil) between April 2016 and November 2019. We included all the results obtained during this period. Patients from geographical regions other than the South were excluded. We calculated the absolute frequency of positive and negative cases. In addition, we calculated the number of cases confirmed by PCR among those with positive IgM antibodies. The results were obtained from a spreadsheet designed to preserve patient confidentiality.

## RESULTS

We recorded 1,189 Zika virus serologies (IgM). Among them, 1,171 (98.5%) yielded negative results, 9 (0.75%) were positive, and 9 (0.75%) were inconclusive. In all 21 RT-PCR tests performed, the results were all negative.

The 9 positive serological tests belonged to 7 different patients. Four of them also had RT-PCR tests done (with negative results), one was serologically re-tested and had an inconclusive result, and two patients didn’t return after the result.

The nine serologies with inconclusive results also belonged to different patients. Four of them (one mentioned above) did not have follow-up tests after their inconclusive results; four had the tests repeated after 25 days and obtained negative results, and one had an RT-PCR test done after 29 days with a negative result as well.

## DISCUSSION

Zika virus spread throughout Brazil until February 2017, and more than 70,000 cases were reported. However, in 2016, the incidences of the infection in the southern regions were drastically lower (as low as 16.8 cases per 100,000 inhabitants) (Brazilian Ministry of Health, 2017).

In addition, in 2016, more than 10,000 children with developmental changes possibly associated to Zika virus infection were reported throughout Brazil. However, the southern regions reported the least number of cases (only 2.2%) (Brazilian Ministry of Health, 2017). Research conducted in a clinic in the southeast of Brazil, with patients undergoing fertility treatments similar to those in our study, found a similar proportion of positive IgM serologies (1.3%). The study also emphasizes that in Brazil, health insurance does not reimburse the money spent by patients for Zika virus screening. The cost of testing is equivalent to 1.2 out of 8 days of ovarian stimulant drug treatment. Therefore, there is financial burden for these patients (Souza *et al*., 2016).

In addition to the financial issues imposed by Zika virus screening, the tests in asymptomatic patients may significantly delay the start of infertility treatments because screening should be repeated within a minimum of 25 days to begin a new cycle of treatment. In some cases, the delay in starting treatment can lead to worse results and greater anxiety for the couple undergoing assisted reproduction.

The study has limitations related to its population and sample characteristics. We collected the data retrospectively, and the examination results were obtained from patients assisted in a private clinic. Considering that infection with Zika virus is correlated to important social factors, the analyzed population may not faithfully reflect the general population.

## Conclusion

Our results indicated a low rate of positive tests, which is consistent with the results of other studies. Therefore, our results make us challenge the validity of Zika virus screening for asymptomatic patients, particularly in low-incidence regions. Compulsory screening may lead to increased anxiety about the fertility treatment with possible poorer outcomes due to delayed therapy initiation and increased financial burden.
